# Adaptive Distributed Student’s T Extended Kalman Filter Employing Allan Variance for UWB Localization

**DOI:** 10.3390/s25061883

**Published:** 2025-03-18

**Authors:** Yanli Gao, Maosheng Yang, Xin Zang, Lei Deng, Manman Li, Yuan Xu, Mingxu Sun

**Affiliations:** 1 Qingdao Branch of Naval Aviation University, Qingdao 266000, China; gaoyanli@stu.ouc.edu.cn; 2 Faculty of Information Science and Engineering, Ocean University of China, Qingdao 266000, China; 3 Shandong Huichuang Information Technology Co., Ltd., Linyi 276002, China; cse_xuy@ujn.edu.cn; 4 School of Electrical Engineering, University of Jinan, Jinan 250022, China; yangms17@stu.ujn.edu.cn (M.Y.); 202321201003@stu.ujn.edu.cn (X.Z.); 5 Jinan Chenhe Information Technology Co., Ltd., Jinan 250022, China; 6 School of Electrical Engineering, Shandong Huayu University of Technology, Dezhou 253034, Chinalmm@huayu.edu.cn (M.L.)

**Keywords:** t distribution, extended Kalman filter, UWB

## Abstract

This study proposes an adaptive distributed Student’s t extended Kalman filter (EKF) using Allan variance for ultrawide-band (UWB) localization. First of all, we model the state equation using the target’s position and velocity in east and north directions and the measurement equation by using distance between the UWB base station (BS) and the target object. Then, the adaptive distributed filter employs a federation structure: A local t EKF is designed to estimate the target’s position by fusing the distance between the UWB base station and the target object. The main filter fuses the local filter’s outputs and computes the final output. For the local t EKF, in order to overcome the problem that noise in the Kalman method is assumed to be white noise and difficult to adapt to practical application environments, the t distribution is used to model noise. Meanwhile, Allan variance is calculated to assist the local filter, which improves the adaptive ability. Experimental results show that the proposed method effectively enhances navigation accuracy compared to the distributed EKF.

## 1. Introduction

As quality-of-life indices have notably risen, the sophistication and refinement of services have increased accordingly. This trend has created new challenges for service robots [1], requiring continuous advancements to meet the evolving needs of various industries. Navigation and positioning, essential for service robots to perform tasks, have gradually become key areas of focus in this research field [2]. Cutting-edge navigation and positioning methods have emerged as a central focus, drawing considerable interest from both academics and industry professionals. Advancements in this domain are expected to significantly enhance the efficiency and reliability of service robots across different sectors. Therefore, ongoing research into innovative approaches for addressing the complex challenges of navigation and positioning is crucial.

Numerous navigation technologies and data fusion methods have been developed to provide continuous and stable navigation for service robots. The widespread adoption of Global Navigation Satellite Systems (GNSSs) is evident across industries like transportation, agriculture, telecommunications, and disaster response. This growth has been fueled by advancements in satellite technology, the widespread availability of GNSS devices, and the seamless integration of GNSS data into various systems and software. For instance, in [3], a two-stage image processing scheme based on the target’s translational motion was explored using GNSS-based passive radar. This method proved effective in tracking three freighters illuminated by multiple satellites. The third-generation Beidou system (BDS-3) uses an extended Kalman filter (EKF) to estimate satellite orbits and timing parameters, employing a dual set of standalone filters for seamless real-time functionality [4]. In [5], the authors examined the contribution of the small Tianjin University-1 (TJU-1) satellite, which uses the global positioning system (GPS) for real-time navigation, alongside BDS-2, GLONASS, and BDS-3, for the first time. While many practical applications based on GNSSs have been proposed, GNSS methods provide stable solutions in outdoor environments. However, GNSS positioning accuracy significantly decreases in certain enclosed environments due to signal interference. Various attempts have been made to address this issue. For example, in [6], inertial-assisted, unmodulated visible-light positioning was used to provide stable localization in environments with weak GNSS signals, demonstrating the method’s efficacy. In [7], radio frequency identification (RFID) technology was proposed for navigation. In this study, a radio frequency fingerprint was developed, with the support of a Kalman filter, to assist localization. A portable, user-controlled device was used to estimate the distance and angle to the target, guiding the user to the intended RFID-tagged target [8]. In [9], a novel UHF RFID tag localization method based on phase measurements across a synthetic aperture was introduced, generating a holographic image to illustrate the spatial probability density function of the tag’s actual position. In [10], ultrawide-band (UWB) technology was used to determine robot coordinates, with a test conducted in a challenging indoor environment. A novel method for non-line-of-sight (NLOS) identification and mitigation using a multi-input parallel deep learning model and a Grampian angular field (GAF) was proposed in [11]. In [12], a novel static person (SP) localization (SPL) method was introduced, featuring an innovative SP detection scheme and integration with SP tracking, offering low computational complexity. However, it is worth noting that these methods require a base station, increasing overall system cost. There are many different approaches to solve localization in indoor environments, e.g., visual SLAM, LiDAR SLAM, etc. It should be noted that these methods are able to build the map and provide the target object’s position; however, the high-precision position information of these methods relies on high-density point cloud information, which results in a relatively high computational load and a long time required for position information calculation. Moreover, if these methods cannot obtain enough feature points, these methods cannot obtain accurate position information. The UWB method can provide position information just by employing several BSs; when compared with visual SLAM and LiDAR SLAM, its cost is cheap. Its localization accuracy is higher than that of similar methods, such as RFID, Wi-Fi, etc. It should be pointed out that all the methods mentioned above are not suitable for all environments.

The integration of cutting-edge technologies, particularly in data processing and sensor systems, has significantly improved data fusion filtering techniques, leading to enhanced localization accuracy. By leveraging various data inputs, these advanced methods offer a more detailed understanding of spatial location [13]. Today, the Kalman filter is the most widely used technique, with its improved versions playing a critical role across various sectors [14]. For example, in [15], a dual-rate Kalman filter (DRKF) combined a time-differential GPS carrier phase and GPS pseudorange with INS measurements. A federated Kalman filter was used to analyze the effects of varying weight distribution in multiple GNSS time-transfer measurements [16]. In [17], an adaptive Kalman filter was employed for GNSS/IMU/LiDAR fusion localization. It is important to note that the Kalman filter is well-suited for linear systems, while the EKF is recommended for nonlinear system dynamics. For example, in [18], an adaptive-network fuzzy inference system (ANFIS)-improved EKF method was proposed for the determination of the accurate positions of autonomous underwater vehicles (AUVs) using a single beacon. In [19], the integration of the nonlinear autoregressive exogenous model (NARX) with an EKF was used to enhance UAV swarm localization. However, the Kalman filters mentioned above primarily assume Gaussian noise, meaning that their performance depends heavily on model accuracy and noise characteristics. In practical applications, noise is often non-Gaussian, making Kalman filters less effective in harsh localization environments.

To address this, this study proposes an adaptive distributed Student’s t EKF using Allan variance for UWB localization. The adaptive distributed filter employs a federation structure. The local filter uses a t EKF to estimate the signal distance between the UWB base station (BS) and the target, modeling the noise with a t distribution. The main filter then fuses the local filter’s outputs to compute the final result, while Allan variance assists the local filter. Experimental results show that the proposed method significantly improves navigation accuracy compared to the distributed EKF.

**Figure 1:**
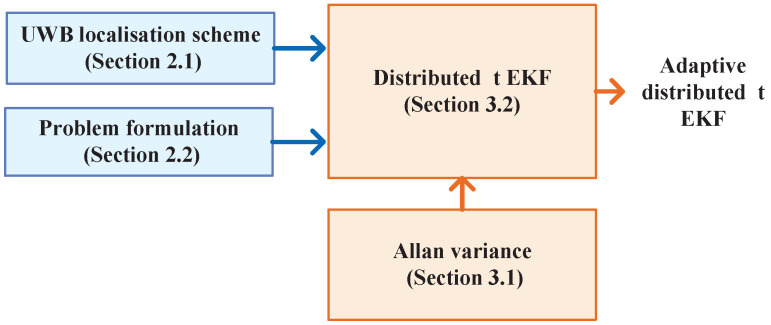
The structure of this paper.

The structure of this paper is shown in Figure 1. The results of our research are summarized as follows:

- We design a distributed UWB localization strategy for mobile objects, utilizing a distributed filter structure. The system employs multiple local filters to estimate the target’s position by fusing distance measurements. The main filter then combines these outputs to compute the final position.
- An adaptive Allan variance computation method was derived, where Allan variance is calculated based on noise estimation from the previous moment.
- Building on the distributed UWB localization scheme and Allan variance method, we propose the adaptive distributed Student’s t EKF. This method uses a t distribution to model the noise, and the main filter fuses the local filter outputs to compute the final result, with Allan variance assisting the local filter.
- Experimental results, supported by rigorous statistical analyses, demonstrate the superior efficiency and effectiveness of the proposed algorithms, which significantly outperform traditional methods.

The remaining sections of this paper are organized as follows: Section 2 presents the problem formulation. Section 3 outlines the adaptive distributed Student’s t EKF. Section 4 evaluates the method’s performance through experimental analysis. Section 5 concludes the paper.

## 2. Problem Formulation

In this section, we design the UWB localization with the adaptive distributed Student’s t EKF strategy and define the related problem.

### 2.1. UWB Localization with the Adaptive Distributed Student’s T Extended Kalman Filter Scheme

**Figure 2:**
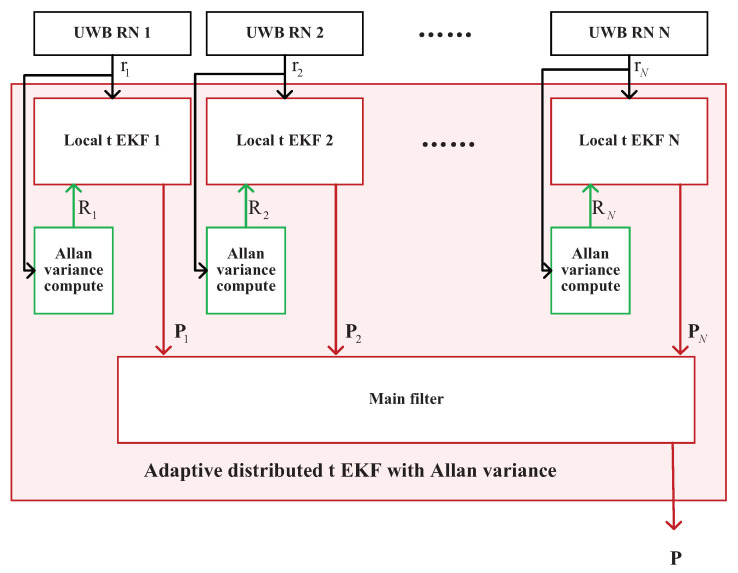
The structure of the UWB localization with the adaptive distributed student’s t extended Kalman filter with Allan variance.

We begin by introducing the UWB localization scheme with the adaptive distributed Student’s t EKF, as shown in Figure 2. The adaptive filter employs a distributed filter structure, consisting of *N* Allan variance-assisted local t EKFs. Each local filter estimates the target object’s position $P_{j}$, j∈[1,N], by fusing the distance measurements $r_{j},j\in \left[1,N\right]$. The main filter then combines the outputs of the local filters to compute the final position of the target. The design of the local Allan variance-assisted t EKF filter and the data fusion model used by the local filters will be discussed in the next section.

### 2.2. Problem Formulation

For Student’s t distribution, if the variable $s_{t}$ follows a t distribution, it is denoted as $s_{t}∼St\left(\mu ,\delta ,\eta \right)$, where μ is the mean, δ is the scale matrix, and η is the degrees of freedom (DOFs). According to [20], the probability density function (pdf) of St(μ,δ,η) is given by Equation (1).

(1) $$\begin{array}{ccc} p\left(s\right) & = & \frac{\Gamma \left(\frac{\eta +2}{2}\right)}{\Gamma \left(\frac{\eta }{2}\right)}\frac{1}{{\left(\eta \pi \right)}^{\frac{m}{2}}}\frac{1}{\sqrt{\det\left(\delta \right)}}{\left\{1+\frac{{\left(s-\mu \right)}^{T}\delta^{-1}\left(s-\mu \right)}{\eta }\right\}}^{-\frac{\eta +2}{2}}\\ & = & \frac{\Gamma \left(\frac{\eta +2}{2}\right)}{\Gamma \left(\frac{\eta }{2}\right)}\frac{1}{{\left(\eta \pi \right)}^{\frac{m}{2}}}\frac{1}{\sqrt{\det\left(\delta \right)}}{\left\{1+\frac{\Lambda^{2}}{\eta }\right\}}^{-\frac{\eta +2}{2}} \end{array}\;,$$

where *m* represents the size of the $s_{t}$ dimension. The equation representing the state for the *i*th local adaptive Student’s t EKF is as follows:

(2) $$\underset{s_{t}^{\left(i\right)-}}{\underset{︸}{\left[\begin{array}{c} x_{t}^{\left(i\right)-}\\ {vx}_{t}^{\left(i\right)-}\\ y_{t}^{\left(i\right)-}\\ {vy}_{t}^{\left(i\right)-} \end{array}\right]}}=\underset{A}{\underset{︸}{\left[\begin{array}{cccc} 1 & 0 & \Delta t & 0\\ 0 & 1 & 0 & \Delta t\\ 0 & 0 & 1 & 0\\ 0 & 0 & 0 & 1 \end{array}\right]}}\underset{s_{t}^{\left(i\right)}}{\underset{︸}{\left[\begin{array}{c} x_{t}^{\left(i\right)}\\ {vx}_{t}^{\left(i\right)}\\ y_{t}^{\left(i\right)}\\ {vy}_{t}^{\left(i\right)} \end{array}\right]}}+\omega_{t}^{\left(i\right)}\;,i\in \left[1,N\right],$$

where $\left(x_{t}^{\left(i\right)},y_{t}^{\left(i\right)}\right)$ denotes the target object’s position information at the time index *t* estimated by the *i*th local filter, $\left({vx}_{t}^{\left(i\right)},{vy}_{t}^{\left(i\right)}\right)$ denotes the target object’s velocity information at the time index *t* estimated by the *i*th local filter, and $\omega_{t}^{\left(i\right)}∼St\left(0,Q_{t}^{\left(i\right)},\eta_{1,t}^{\left(i\right)}\right)$ is the system noise under Student’s t distribution. Here, $Q_{t}^{\left(i\right)}$ is the scale matrix for the *i*th local filter, and $\eta_{1,t}^{\left(i\right)}$ is the DOFs.

(3) $$r_{t}^{\left(i\right)}=\underset{g\left(s_{t}^{\left(i\right)-}\right)}{\underset{︸}{\sqrt{{\left(x_{t}^{\left(i\right)-}-\left(x_{R}^{\left(i\right)}\right)\right)}^{2}+{\left(y_{t}^{\left(i\right)-}-\left(y_{R}^{\left(i\right)}\right)\right)}^{2}}}}+v_{t}^{\left(i\right)}\;,i\in \left[1,N\right],$$

where $r_{t}^{\left(i\right)}$ means the range from the *i*th UWB BS to the target object, $\left(x_{R}^{\left(i\right)},y_{R}^{\left(i\right)}\right)$ is the *i*th UWB BS’s position, and the $v_{t}^{\left(i\right)}∼St\left(0,R_{t}^{\left(i\right)},\eta_{2,t}^{\left(i\right)}\right)$ is the measurement noise under Student’s t distribution, Here, $R_{t}^{\left(i\right)}$ is the scale matrix for the *i*th local filter, and $\eta_{2,t}^{\left(i\right)}$ is the DOFs.

## 3. Adaptive Distributed Student’s T EKF

In this part, we detail the design of the adaptively distributed Student’s t EKF according to Figure 2. Firstly, we introduce Allan variance based on the model (2) and (3). Next, the distributed Student’s t EKF is designed. Finally, the Allan variance-assisted method is proposed.

### 3.1. Allan Variance

As one of the most widely used time-series analysis method, Allan variance is proposed to extract noise from data [6]. In this work, we modify the traditional definition of Allan variance to provide adaptive noise estimation. Starting with the traditional definition of Allan variance, with $r^{\left(i\right)}$, we can obtain that

(4) $$\sigma_{r^{\left(i\right)}}^{2}=\frac{1}{2}E\left({\left(r_{j+1}^{\left(i\right)}\left(t\right)-r_{j}^{\left(i\right)}\left(t\right)\right)}^{2}\right)\;,i\in \left[1,N\right],$$

where *j* is the number of data groups. When the time index is $t_{0}$, *N* is the number of UWB BSs. We can obtain that

(5) $${\hat{R}}_{t_{0}}^{\left(i\right)}=\frac{1}{2\left(t_{0}-1\right)}\sum_{j=1}^{N-1}\left[{\left(r_{i+1}^{\left(i\right)}\left(\delta \right)-r_{i}^{\left(i\right)}\left(\delta \right)\right)}^{2}\right]\;,i\in \left[1,N\right],$$

where *N* is the sum of the data groups. Then, we can modify this equation:

(6) $$\begin{array}{ccc} {\hat{R}}_{t}^{\left(i\right)} & = & \frac{1}{2\left(t-1\right)}\sum_{j=2}^{t}\left[{\left(r_{j}^{\left(i\right)}-r_{j-1}^{\left(i\right)}\right)}^{2}\right]\\ & = & \frac{1}{2\left(t-1\right)}\left\{\sum_{j=2}^{t-1}\left[{\left(r_{j}^{\left(i\right)}-r_{j-1}^{\left(i\right)}\right)}^{2}\right]+{\left(r_{t}^{\left(i\right)}-r_{t-1}^{\left(i\right)}\right)}^{2}\right\}\\ & = & \frac{t-2}{t-1}\left\{\frac{1}{2\left(t-1\right)}\sum_{j=2}^{t}\left[{\left(r_{j}^{\left(i\right)}-r_{j-1}^{\left(i\right)}\right)}^{2}\right]\right\}+\frac{1}{2\left(t-1\right)}{\left(r_{t}^{\left(i\right)}-r_{t-1}^{\left(i\right)}\right)}^{2}\\ & = & \left(1-\frac{1}{t-1}\right){\hat{R}}_{t-1}^{\left(i\right)}+\frac{1}{2\left(t-1\right)}{\left(r_{t}^{\left(i\right)}-r_{t-1}^{\left(i\right)}\right)}^{2}\;,i\in \left[1,N\right],t\geq 2, \end{array}$$

Thus, we can obtain

(7) $${\hat{R}}_{t}^{\left(i\right)}=\left(1-\chi_{t-1}\right){\hat{R}}_{t-1}^{\left(i\right)}+\frac{1}{2}\chi_{t-1}{\left(r_{t}^{\left(i\right)}-r_{t-1}^{\left(i\right)}\right)}^{2}\;,i\in \left[1,N\right],$$

In this work, we modify Equation (7) as follows:

(8) $${\hat{R}}_{t}^{\left(i\right)}=\left\{\begin{array}{cc} \left(1-\chi_{t-1}\right){\hat{R}}_{t-1}^{\left(i\right)}+\chi_{t-1}{\hat{R}}_{\min}^{\left(i\right)}, & {\hat{R}}_{t}^{\left(i\right)}<{\hat{R}}_{\min}^{\left(i\right)}\\ {\hat{R}}_{\max}^{\left(i\right)}, & {\hat{R}}_{t}^{\left(i\right)}>{\hat{R}}_{\max}^{\left(i\right)}\\ \left(1-\chi_{t-1}\right){\hat{R}}_{t-1}^{\left(i\right)}+\frac{1}{2}\chi_{t-1}{\left(r_{t}^{\left(i\right)}-r_{t-1}^{\left(i\right)}\right)}^{2}, & otherwise \end{array}\right.$$

where ${\hat{R}}_{\max}^{\left(i\right)}$ and ${\hat{R}}_{\min}^{\left(i\right)}$ represent the defined maximum and minimum values of $\hat{R}$, respectively.

### 3.2. Distributed Student’s T EKF

Within this subsection, we will derive the distributed Student’s t EKF, utilizing the model (2) and (3).

In this work, we first design the following joint density:

(9) $$p\left(s_{t}^{\left(i\right)},v_{t}^{\left(i\right)}|r_{1:t}^{\left(i\right)}\right)=St\left(\left[\begin{array}{c} s_{t}^{\left(i\right)}\\ v_{t}^{\left(i\right)} \end{array}\right];\left[\begin{array}{c} {\hat{s}}_{t}^{\left(i\right)}\\ 0 \end{array}\right],\left[\begin{array}{cc} {\hat{P}}_{t}^{\left(i\right)} & 0\\ 0 & Q_{t}^{\left(i\right)} \end{array}\right],\eta_{3,t}\right)\;,$$

With the EKF, we can obtain the one-step estimation as follows:

(10) $${\hat{s}}_{t}^{\left(i\right)-}={A\hat{s}}_{t}^{\left(i\right)}\;,$$

(11) $${\hat{P}}_{t}^{\left(i\right)-}={A\hat{P}}_{t}^{\left(i\right)}A^{T}+Q_{t}^{\left(i\right)}\;,$$

$\eta_{3,t}$ is retained, and we obviously recover the EKF time update. Next, we consider the following joint pdf:

(12) $$p\left(s_{t}^{\left(i\right)},v_{t}^{\left(i\right)}|r_{1:t-1}^{\left(i\right)}\right)=St\left(\left[\begin{array}{c} s_{t}^{\left(i\right)}\\ v_{t}^{\left(i\right)} \end{array}\right];\left[\begin{array}{c} {\hat{s}}_{t}^{\left(i\right)-}\\ 0 \end{array}\right],\left[\begin{array}{cc} {\hat{P}}_{t}^{\left(i\right)-} & 0\\ 0 & R_{t}^{\left(i\right)} \end{array}\right],\eta_{3,t-1}\right)\;,$$

Then, we can compute the following joint pdf:

(13) $$p\left(s_{t}^{\left(i\right)},v_{t}^{\left(i\right)}|r_{1:t-1}^{\left(i\right)}\right)=St\left(\left[\begin{array}{c} s_{t}^{\left(i\right)}\\ r_{t}^{\left(i\right)} \end{array}\right];\left[\begin{array}{c} {\hat{s}}_{t}^{\left(i\right)-}\\ G_{t}^{\left(i\right)}{\hat{s}}_{t}^{\left(i\right)-} \end{array}\right],\left[\begin{array}{cc} {\hat{P}}_{t}^{\left(i\right)-} & {\hat{P}}_{t}^{\left(i\right)-}{\left(G_{t}^{\left(i\right)}\right)}^{T}\\ G_{t}^{\left(i\right)}{\hat{P}}_{t}^{\left(i\right)-} & \underset{S_{t}^{\left(i\right)}}{\underset{︸}{G_{t}^{\left(i\right)}{\hat{P}}_{t}^{\left(i\right)-}{\left(G_{t}^{\left(i\right)}\right)}^{T}+R_{t}^{\left(i\right)}}} \end{array}\right],\eta_{3,t-1}\right)\;,$$

where $G_{t}^{\left(i\right)}=\frac{\partial g\left(s_{t}^{\left(i\right)-}\right)}{\partial s_{t}^{\left(i\right)}}$. The updated parameters of the t EKF can be computed by the following equations:

(14) $$\eta_{3,t}=\eta_{3,t-1}+m_{r_{t}^{\left(i\right)}}\;,$$

where $m_{r_{t}^{\left(i\right)}}$ is the dimension of $r_{t}^{\left(i\right)}$.

(15) $${\hat{s}}_{t}^{\left(i\right)}={\hat{s}}_{t}^{\left(i\right)-}+{\hat{P}}_{t}^{\left(i\right)-}{\left(G_{t}^{\left(i\right)}\right)}^{T}{\left(G_{t}^{\left(i\right)}{\hat{P}}_{t}^{\left(i\right)-}{\left(G_{t}^{\left(i\right)}\right)}^{T}+R_{t}^{\left(i\right)}\right)}^{-1}\left(r_{t}^{\left(i\right)}-g\left(s_{t}^{\left(i\right)-}\right)\right)\;,$$

(16) $${\hat{P}}_{t}^{\left(i\right)}=\frac{\eta_{3,t-1}+\Lambda_{r_{t}^{\left(i\right)}}^{2}}{\eta_{3,t-1}+m_{r_{t}^{\left(i\right)}}}\left[{\hat{P}}_{t}^{\left(i\right)-}-{\hat{P}}_{t}^{\left(i\right)-}{\left(G_{t}^{\left(i\right)}\right)}^{T}{\left(G_{t}^{\left(i\right)}{\hat{P}}_{t}^{\left(i\right)-}{\left(G_{t}^{\left(i\right)}\right)}^{T}+R_{t}^{\left(i\right)}\right)}^{-1}G_{t}^{\left(i\right)}{\hat{P}}_{t}^{\left(i\right)-}\right]\;,$$

(17) $$\Lambda_{r_{t}^{\left(i\right)}}^{2}={\left(r_{t}^{\left(i\right)}-g\left(s_{t}^{\left(i\right)-}\right)\right)}^{T}{\left(G_{t}^{\left(i\right)}{\hat{P}}_{t}^{\left(i\right)-}{\left(G_{t}^{\left(i\right)}\right)}^{T}+R_{t}^{\left(i\right)}\right)}^{-1}\left(r_{t}^{\left(i\right)}-g\left(s_{t}^{\left(i\right)-}\right)\right)\;,$$

With ${\hat{X}}_{t}^{\left(i\right)}$ and ${\hat{P}}_{t}^{\left(i\right)}$, we can output the final output of the filter using the following equations:

(18) $${\hat{P}}_{t}={\left({\hat{P}}_{t}^{\left(1\right)}\right)}^{-1}+{\left({\hat{P}}_{t}^{\left(2\right)}\right)}^{-1}+\cdots +{\left({\hat{P}}_{t}^{\left(N\right)}\right)}^{-1}\;,$$

(19) $${\hat{s}}_{t}={\hat{P}}_{t}\left[{\left({\hat{P}}_{t}^{\left(1\right)}\right)}^{-1}{\hat{s}}_{t}^{\left(1\right)}+{\left({\hat{P}}_{t}^{\left(2\right)}\right)}^{-1}{\hat{s}}_{t}^{\left(2\right)}+\cdots +{\left({\hat{P}}_{t}^{\left(N\right)}\right)}^{-1}{\hat{s}}_{t}^{\left(N\right)}\right]\;,$$

### 3.3. Adaptive Distributed Student’s T Filter

Using Allan variance and the distributed Student’s t EKF, we can now introduce our adaptive distributed Student’s t EKF, as outlined in the following algorithm. From Algorithm 1, we can see that, firstly, we employ the local t EKF to estimate the target object’s position by fusing $r_{t}^{\left(i\right)}$ (lines 4–10); then, we compute ${\hat{R}}_{t}^{\left(i\right)}$ by Equation (9) (line 11). With ${\hat{X}}_{t}^{\left(i\right)}$ and ${\hat{P}}_{t}^{\left(i\right)}$, we can then obtain the final output of the filter.
**Algorithm 1:** Adaptive distributed Student’s t extended Kalman filter for model (2) and (3)
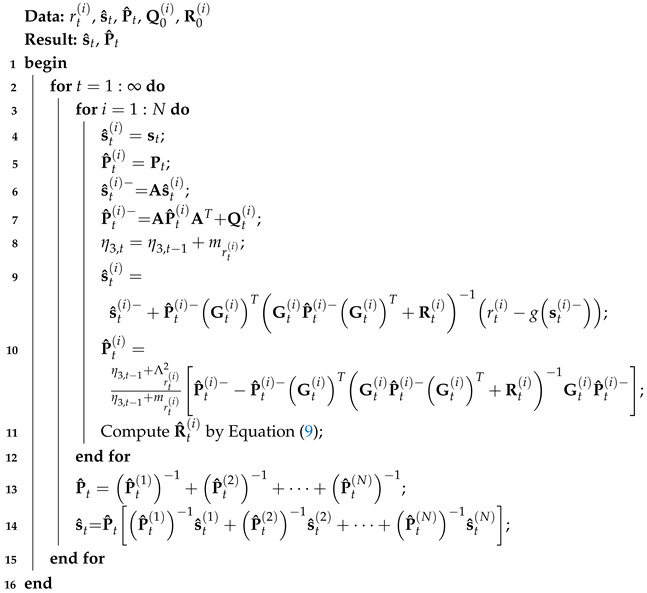


## 4. Test and Discussion

This section presents two practical tests to prove the efficacy of our proposed method. One test involved UWB mobile robot localization, while the other focused on UWB robotic dog localization.

### 4.1. UWB Mobile Robot Localization

**Figure 3:**
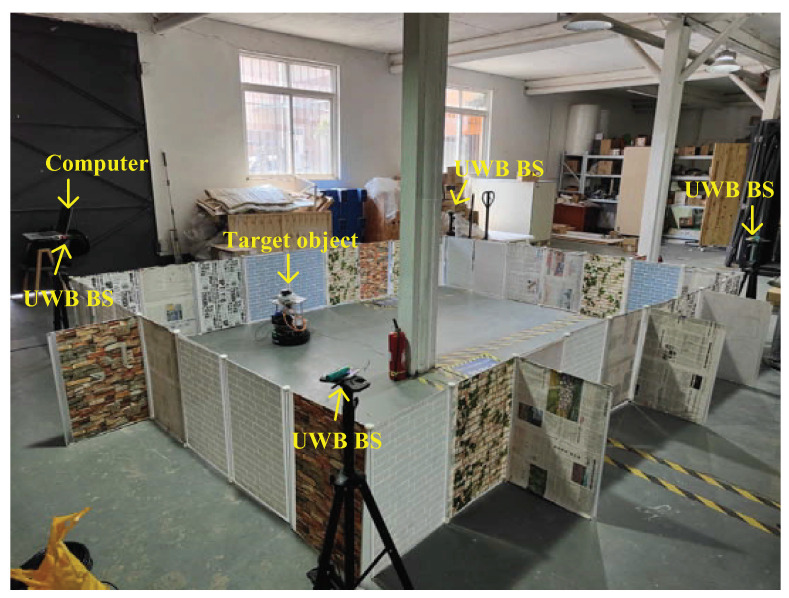
Environment for UWB mobile robot localization.

**Figure 4:**
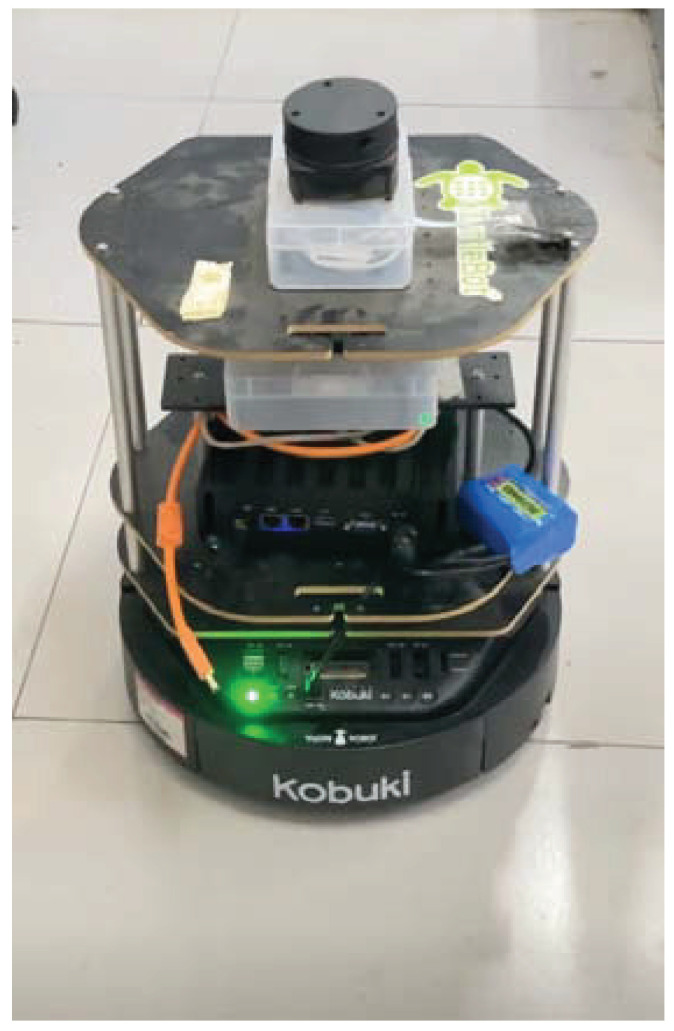
The mobile robot used in the test.

The first test involved UWB mobile robot localization, In this test, we employed four UWB BSs placed in known preset positions. The UWB blind node (BN) was attached to the target object, which in this case was the mobile robot. The test environment is shown in Figure 3. We can observe that the conditions were harsh: First of all, there were two obvious metal pillars in this scene. Secondly, the testing scenario was quite chaotic due to the accumulation of goods. The above factors led to the signal of the UWB positioning being affected; thus, we can determine that the conditions were harsh in this test.The mobile robot used in this test is shown in Figure 4. For comparison, we employed the UWB solution, federal EKF, and distributed t EKF as benchmarks. For the filter, we set $s_{0}={\left[\begin{array}{cccc} -0.2 & 0 & 0.1 & 0 \end{array}\right]}^{T}$, $P=I_{4\times 4}$, $Q=\left[\begin{array}{cccc} 0.0002 & 0.0034 & 0 & 0\\ 0.0034 & 0.0676 & 0 & 0\\ 0 & 0 & 0.0002 & 0.0034\\ 0 & 0 & 0.0034 & 0.0676 \end{array}\right]$, R=0.0032.

**Figure 5:**
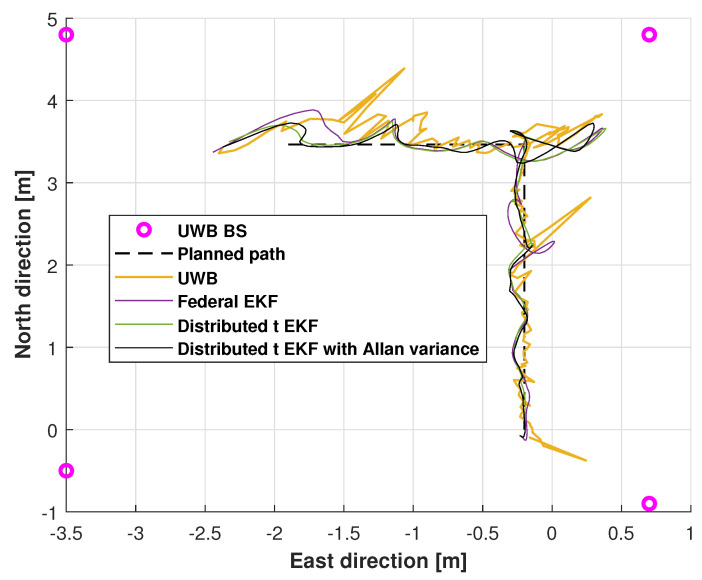
The planned path, path of UWB measurements, and paths measured by the federal EKF, distributed t EKF, and adaptive distributed t EKF with Allan variance for the mobile robot localization.

Figure 5 shows the planned path, the UWB measurement path, the path from the federal EKF, and the paths from the distributed t EKF and adaptive distributed t EKF with Allan variance for mobile robot localization. In the figure, the planned path is represented by a black dashed line, the UWB computed path by a blue solid line (calculated using the least-squares algorithm), the federal EKF path by an orange solid line, the distributed t EKF path by a yellow solid line, and the adaptive distributed t EKF with Allan variance path by a black solid line. The UWB BSs are marked by pink circles. As seen in Figure 5, the UWB estimated trajectory exhibits significant fluctuations, indicating that the complex indoor environment affected the positioning accuracy of the least-squares method. In contrast, while the federal EKF path also shows fluctuations, its deviations are larger than those in the UWB solution. The distributed t EKF and the proposed adaptive distributed t EKF with Allan variance closely follow the reference path, demonstrating superior accuracy compared to both the UWB and federal EKF methods.

**Figure 6:**
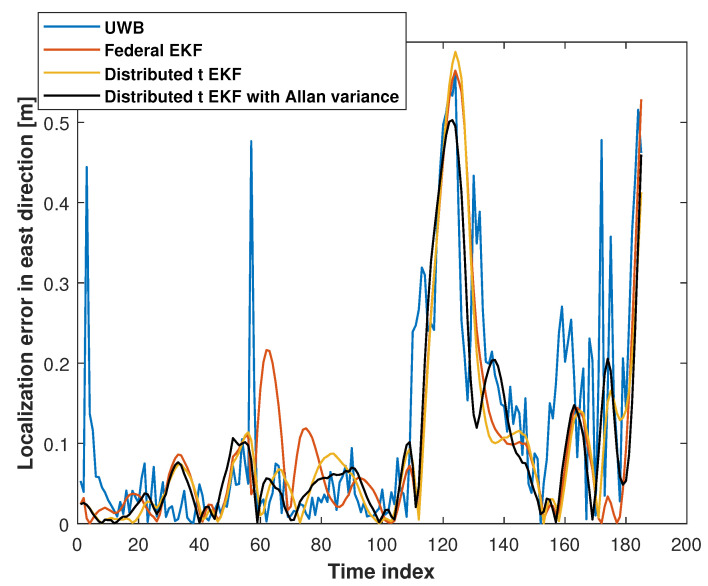
The east absolute position error measured by the UWB, federal EKF, distributed t EKF, and adaptive distributed t EKF with Allan variance for the mobile robot localization.

**Figure 7:**
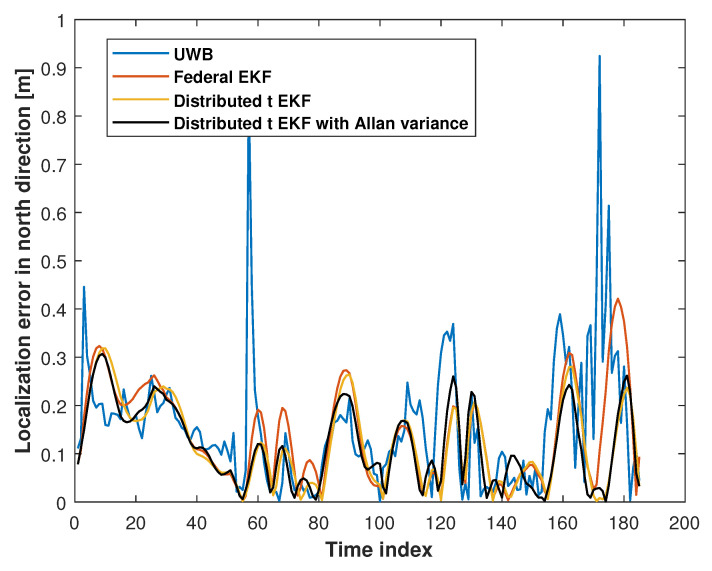
The north absolute position error of the UWB measurements, federal EKF, distributed t EKF, and adaptive distributed t EKF with Allan variance for the mobile robot localization.

Figure 6 and Figure 7 show the eastward and northward absolute position errors for the UWB measurements, federal EKF, distributed t EKF, and adaptive distributed t EKF with Allan variance for mobile robot localization. In the eastward direction, the UWB solution exhibited noticeable jitter errors, particularly between the time indices 0 and 20, 60 and 65, and 100 and 150, indicating that the harsh environment affected UWB localization accuracy. The federal EKF reduced some of these errors but still showed residual inaccuracies, such as between the time indices 50 and 80. The distributed t EKF could reduce the localization error further when compared with the federal EKF. In contrast, the adaptive distributed t EKF with Allan variance further reduced the error, demonstrating the advantages of the t distribution under complex noise conditions. In the northward direction, the results of the methods were similar to those in the east direction. The UWB localization still exhibited noticeable jitter errors in the north direction, while the federal EKF exhibited reduced localization error compared to the UWB approach, highlighting the effectiveness of this method in mitigating UWB localization error. The proposed adaptive distributed t EKF with Allan variance outperforms both the UWB and distributed EKF methods.

The root mean square errors (RMSEs) for the UWB measurements, federal EKF, distributed t EKF, and adaptive distributed t EKF with Allan variance are summarized in Table 1. The table shows that the adaptive distributed t EKF reduced the localization error from 0.182 m to 0.145 m in the east direction and from 0.205 m to 0.142 m in the north direction compared to the UWB solution. When compared to the federal EKF method’s mean value in the east and north directions, the proposed method reduced the error by about 13.25%, demonstrating its effectiveness in minimizing localization error.

### 4.2. UWB Robotic Dog Localization

**Figure 8:**
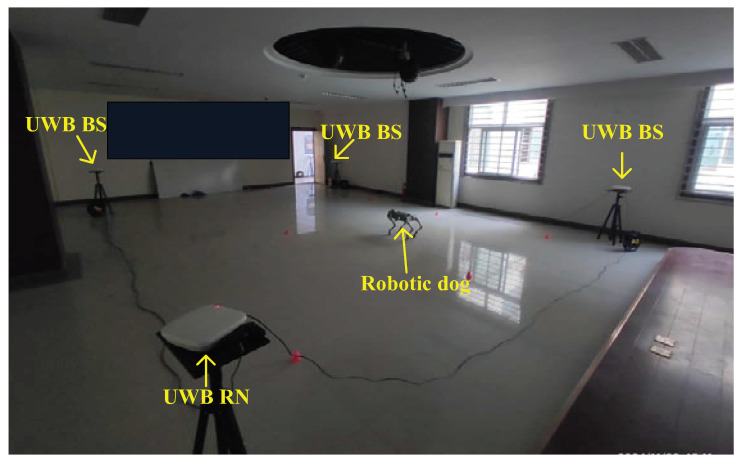
Environment for UWB robotic dog localization.

**Figure 9:**
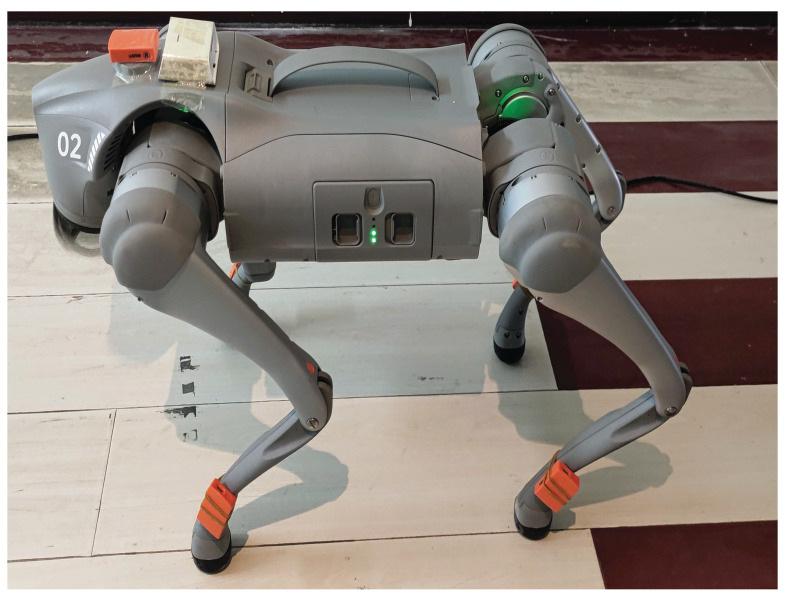
The robotic dog used in the test.

In this subsection, we detail how we employed the UWB, the federal EKF, and the adaptive distributed t EKF with Allan variance to estimate the robotic dog’s position. In this test, we used eight UWB BSs, which are shown in Figure 11. The environment for the UWB robotic dog localization and the robotic dog used in this test are shown in Figure 8 and Figure 9, respectively. We also used the UWB solution and the distributed EKF as reference standards in this test. For the filter, we set $s_{0}={\left[\begin{array}{cccc} 0 & 0 & 0 & 0 \end{array}\right]}^{T}$, $P=I_{4\times 4}$, $Q=\left[\begin{array}{cccc} 00.0000 & 00.0007 & 0 & 0\\ 00.0007 & 00.0144 & 0 & 0\\ 0 & 0 & 00.0000 & 00.0007\\ 0 & 0 & 00.0007 & 00.0144 \end{array}\right]$, R=0.0025.

**Figure 10:**
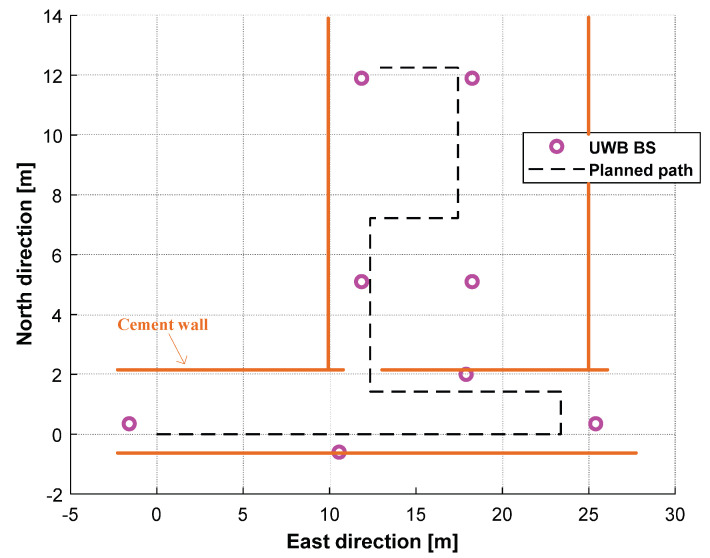
The planned path used by the robotic dog in the test.

**Figure 11:**
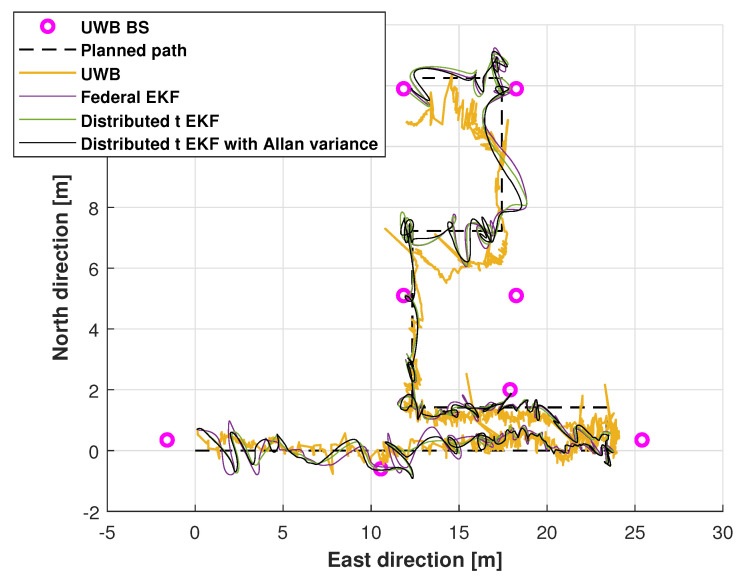
The planned path and the paths of the UWB measurements, federal EKF, distributed t EKF, and adaptive distributed t EKF with Allan variance for the robotic dog localization.

The planned path used by the robotic dog in the test is shown in Figure 10. From the figure, we can see that the cement wall affected the UWB signal, which reduced the localization accuracy of the UWB. The planned path and the trajectory measured by the UWB, the federal EKF, and the adaptive distributed t EKF with Allan variance for the robotic robot localization are shown in Figure 11. From Figure 11, it is clear that the UWB solution exhibited larger errors compared to in the mobile robot test, especially in narrow corridors. When compared with the UWB, both the distributed EKF and the proposed distributed t EKF with Allan variance reduced the localization error, with the proposed method bringing the path closer to the planned path.

**Figure 12:**
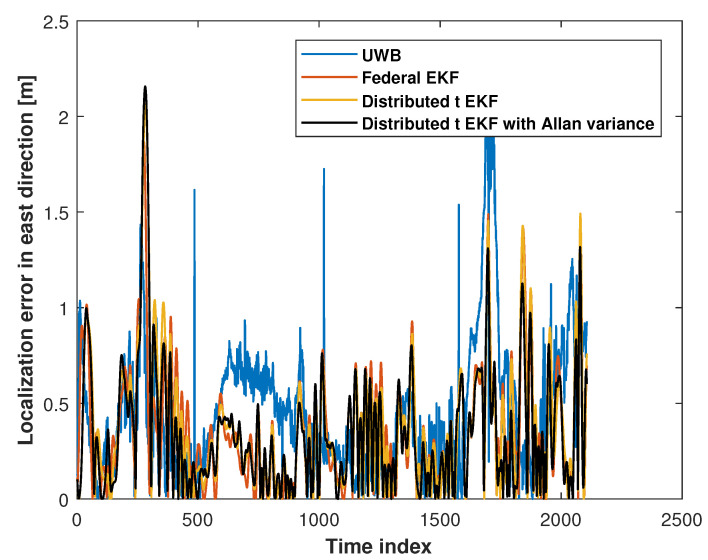
The eastward absolute position error of the UWB measurements, federal EKF, distributed t EKF, and adaptive distributed t EKF with Allan variance for the robotic dog localization.

**Figure 13:**
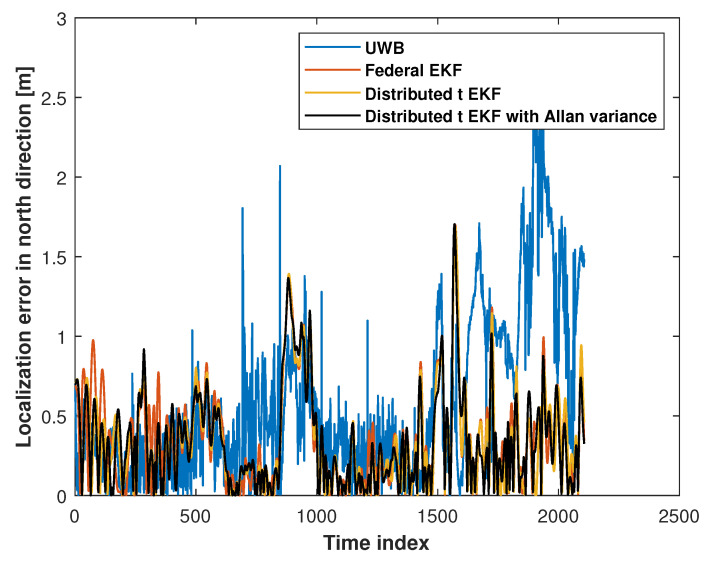
The northward absolute position error measured by the UWB, federal EKF, distributed t EKF, and adaptive distributed t EKF with Allan variance for the robotic dog localization.

Figure 12 and Figure 13 show the east and north absolute position errors for the UWB measurements, federal EKF, and adaptive distributed t EKF with Allan variance in the robotic dog localization test. In these figures, the UWB solution is represented by a continuous blue solid line, the path of the distributed EKF measurements by an orange solid line, and the proposed adaptive distributed t EKF with Allan variance by a black solid line. From the Figure 12, it can be observed that both filters provided stable solutions, while the UWB measurements exhibited large localization errors. This highlights the effectiveness of the filters in improving positional accuracy. Moreover, the proposed adaptive distributed t EKF with Allan variance resulted in smaller localization error compared to the traditional distributed EKF. Thus, it can be concluded that incorporating the t distribution was well suited for this localization environment. From Figure 13, in the north direction, we can see that the filers performed well compared to the UWB, especially in the final stage of the test. It should be noted that the error in the filter solutions was higher than that of the UWB. However, it is important to mention that in this test, the convergence speed of both filters still needed improvement.

The RMSEs measured by the UWB, the federal EKF, the distributed t EKF, and the adaptive distributed t EKF with Allan variance for the robotic dog localization are listed in Table 2. From the table, we can see that the proposed adaptive distributed t EKF with Allan variance method reduced the localization error from 0.568 m to 0.468 m in the east direction and from 0.764 m to 0.451 m in the north directions, respectively, compared to the UWB solutions. Then, we repeated the test again, and the results are listed in Table 3. From the table, we can see that the proposed method had the smallest localization error. Finally, we list the mean values for the two tests in Table 4; we can see that the proposed method had the best performance. Compared to the mean value of the distributed t EKF method, the proposed method reduced the error, demonstrating its effectiveness in minimizing the localization error.

## 5. Conclusions

This study investigates UWB localization using the adaptive distributed Student’s t EKF with Allan variance. The distributed Student’s t EKF is designed as follows: First, a local t EKF for the signal distance between the UWB BS and the target object is proposed, which incorporates the t distribution. Then, the main filter fuses the local filter’s outputs and computes the final result. Meanwhile, the Allan variance is computed to assist the local filter. Experimental results confirm the superiority of the proposed adaptive distributed Student’s t EKF with Allan variance, demonstrating the method’s effectiveness. We are currently investigating the effectiveness of the proposed method in enhancing our understanding of processes affected by colored and interrelated noise patterns. The objective of this research is to meticulously analyze and clarify the complexities of these processes, providing deeper insights into their operational dynamics. With a strong commitment to advancing knowledge, we aim to disseminate the comprehensive findings and inferences derived from these studies. Our intention is to publish these results in the near future, contributing to the growing body of academic research in this specialized field.

## Figures and Tables

**Table 1:** RMSEs measured by the UWB, federal EKF, distributed t EKF, and adaptive distributed t EKF with Allan variance for the mobile robot localization.

Methods	East Position	North Position	Mean
UWB	0.182	0.205	0.194
Federal EKF	0.161	0.170	0.166
Distributed t EKF	0.155	0.146	0.151
Adaptive distributed t EKF with Allan variance	0.145	0.142	0.144

**Table 2:** RMSEs measured by the UWB, federal EKF, distributed t EKF, and adaptive distributed t EKF with Allan variance for the robotic dog localization for the first time.

Methods	East Position	North Position	Mean
UWB	0.568	0.764	0.666
Federal EKF	0.475	0.474	0.476
Distributed t EKF	0.500	0.458	0.479
Adaptive distributed t EKF with Allan variance	0.468	0.451	0.459

**Table 3:** RMSEs measured by the UWB, federal EKF, distributed t EKF, and adaptive distributed t EKF with Allan variance for the robotic dog localization for the second time.

Methods	East Position	North Position	Mean
UWB	0.661	0.613	0.637
Federal EKF	0.624	0.611	0.618
Distributed t EKF	0.644	0.603	0.623
Adaptive distributed t EKF with Allan variance	0.625	0.599	0.612

**Table 4:** RMSEs measured by the UWB, federal EKF, distributed t EKF, and adaptive distributed t EKF with Allan variance for the robotic dog localization.

Methods	First Time	Second Time	Mean
UWB	0.666	0.637	0.652
Federal EKF	0.476	0.618	0.547
Distributed t EKF	0.479	0.623	0.551
Adaptive distributed t EKF with Allan variance	0.459	0.612	0.535

## Data Availability

All related data are contained in this article.

## Abbreviations

This manuscript utilizes the following abbreviations:

ANFIS	Adaptive-Network Fuzzy Inference System
AUVs	Autonomous Underwater Vehicles
BDS	BeiDou Navigation Satellite System
BN	Blind Node
DRKF	Dual-Rate Kalman Filter
DOF	Degrees of Freedom
EKF	Extended Kalman Filter
GAF	Gramian Angular Field
GLONASS	Global Navigation Satellite System
GNSS	Global Navigation Satellite System
GPS	Global Positioning System
LS	Least Squares
RFID	Radio Frequency Identification
RMSE	Root Mean Square Error
SP	Static Person
SPL	Static Person Localization
TJU-1	Tianjin University-1
NARX	Nonlinear Autoregressive Exogenous model
NLOS	Non-Line-Of-Sight
PDF	Probability Density Function
UWB	Ultrawide Band
BS	Base Station
